# Toward a multidimensional fitness paradigm: a critical review and agenda for science, practice, and policy

**DOI:** 10.3389/fpubh.2026.1841661

**Published:** 2026-09-17

**Authors:** Fırat Çağlar Erdem, Türker Bıyıklı, Çağlar Soylu, Görkem Açar

**Affiliations:** 1 Coaching Education Department, School of Physical Education and Sports, Istanbul Nişantaşı University, Istanbul, Türkiye; 2 Department of Coaching Education, Faculty of Sport Sciences, Marmara University, Istanbul, Türkiye; 3 Gulhane Faculty of Physiotherapy and Rehabilitation, University of Health Sciences, Ankara, Türkiye; 4 Faculty of Sport Sciences, Istanbul Gelisim University, Istanbul, Türkiye

**Keywords:** AI ethics, behavior change, biopsychosocial model, digital health, health equity, mental fitness, occupational health, physical fitness

## Abstract

This study aims to critically re-evaluate classical definitions of physical fitness proposed by leading institutions such as the American College of Sports Medicine (ACSM), the World Health Organization (WHO), and the National Academy of Sports Medicine (NASM). Despite their physiological robustness, these definitions no longer capture the multidimensional determinants of human functionality in modern life. The paper introduces a multidimensional fitness paradigm that reframes fitness as a biopsychosocio-emotional capacity integrating physical, mental, social, occupational, and digital dimensions. A narrative critical review of transdisciplinary literature was conducted across the fields of behavioral science, social epidemiology, occupational health, and digital medicine. Conceptual, methodological, and operational gaps in existing frameworks were identified and critically appraised. Drawing from the biopsychosocial model, self-determination theory, behavior change frameworks (COM-B), and digital ethics literature, the study synthesized theoretical foundations and proposed measurable indicators for five interacting dimensions of fitness. Cross-cutting themes such as equity, ethics, data governance, and cultural variability were analyzed to ensure applicability across diverse populations. The review reveals that traditional physiological metrics—such as cardiorespiratory endurance, strength, and body composition—are necessary but insufficient to account for overall human functioning. Evidence demonstrates that cognitive control, emotion regulation, social connectedness, occupational balance, and digital literacy substantially influence wellbeing, resilience, and sustainable performance. The proposed multidimensional model operationalizes fitness through integrated indicators and suggests a validation roadmap encompassing reliability, validity, feasibility, and fairness. Furthermore, illustrative applications in clinical, workplace, and municipal contexts demonstrate how multidimensional fitness can guide personalized interventions and policy innovations. The multidimensional fitness paradigm expands the conceptual boundaries of fitness from a purely physiological construct to an integrative biopsychosocio-digital framework. By embedding ethical, cultural, and technological considerations, it offers a roadmap for 21st-century health promotion and sustainable performance. The study concludes with a 10-point research and policy agenda emphasizing equity, data ethics, and cross-sectoral integration, positioning fitness as both a scientific construct and a societal compass for human flourishing. The framework is presented as a conceptual proposal: its structure, indicators, and applications remain to be empirically validated.

## 1 Introduction

### 1.1 Why the fitness concept needs reframing

For more than half a century, classical definitions of physical fitness have dominated the field. The frameworks codified by the American College of Sports Medicine’s Guidelines for Exercise Testing and Prescription, currently in its 12th edition (1), the World Health Organization’s global action plans (2, 3), and the National Academy of Sports Medicine’s practice manuals (4) have made substantial contributions to the advancement of exercise science, clinical practice, and public health. Their enduring strength lies in operationalizing physiological components—such as cardiorespiratory endurance, muscular strength and endurance, flexibility, and body composition—through standardized protocols. Furthermore, these definitions have clarified the role of modifiable behaviors in reducing cardiometabolic disease risk and enabled the development of testable and scalable prescriptions (3, 5, 6).

Nevertheless, contemporary health and performance extend far beyond physiological indicators. Today, individuals’ functionality and flourishing are significantly shaped by intertwined factors such as stress management, emotion regulation, identity formation, social networks, job design, and digital environments (7, 8). Consequently, fitness has often been equated with exercise capacity, while sustainable daily functioning depends on a much broader set of biopsychosocial resources. The limitation is not that traditional definitions are “incorrect,” but rather that they are “incomplete” for 21 first century contexts characterized by knowledge work, ubiquitous connectivity, and the accelerating integration of human and technological systems (9, 10).

Against this backdrop, the present narrative critical review advances a reframing of fitness as a multidimensional, dynamic, and ethically grounded capacity. Specifically, the review aims to (a) analyze the limitations of legacy definitions, (b) propose a multidimensional fitness model consisting of five interacting dimensions—physical, mental, social, occupational, and digital fitness, (c) outline mechanisms and measurement approaches for each dimension, (d) interrogate cross-cutting issues such as equity, ethics, and cultural variability, and (e) articulate a research and policy agenda to evaluate and implement a holistic fitness paradigm across individuals, organizations, and populations.

### 1.2 Review approach

This article is a narrative critical review, not a systematic review, and no claim of exhaustive or fully reproducible coverage is made. Literature was retrieved through iterative searches of PubMed/MEDLINE, Scopus, Web of Science, and Google Scholar, combining fitness-related terms (e.g., “physical fitness,” “mental fitness,” “social fitness,” “occupational health,” “digital health,” “eHealth literacy”) with terms describing measurement, behavior change, recovery, and ethics. Sources were purposively selected on the basis of conceptual relevance, methodological quality, and disciplinary breadth, prioritizing meta-analyses, consensus statements and guidelines, and seminal theoretical works; backward and forward citation searching supplemented database queries. Because the aim was conceptual synthesis rather than quantitative aggregation, no formal inclusion/exclusion protocol, risk-of-bias assessment, or meta-analytic pooling was undertaken, and wording implying systematic-review methodology has been removed throughout the revised manuscript.

### 1.3 What traditional definitions get right—and what they miss

#### 1.3.1 Strengths of the classical paradigm

Classical definitions of physical fitness provide several enduring strengths that have shaped exercise science and public health for decades. First, they translate into operational components measurable with standardized protocols—for instance, VO₂max, one-repetition maximum (1RM), and field-based performance tests—thus enabling reliable surveillance and prescription across diverse populations (1, 6). Second, these frameworks are underpinned by robust epidemiological evidence linking physical activity to reduced incidence and mortality from noncommunicable diseases, thereby confirming their public health relevance (3, 5). Third, their use of common metrics facilitates comparability across studies and programs, making them indispensable tools in both clinical practice and large-scale population health monitoring. Collectively, these strengths explain why the classical paradigm remains a cornerstone of exercise science (Figure 1).

**Figure 1:**
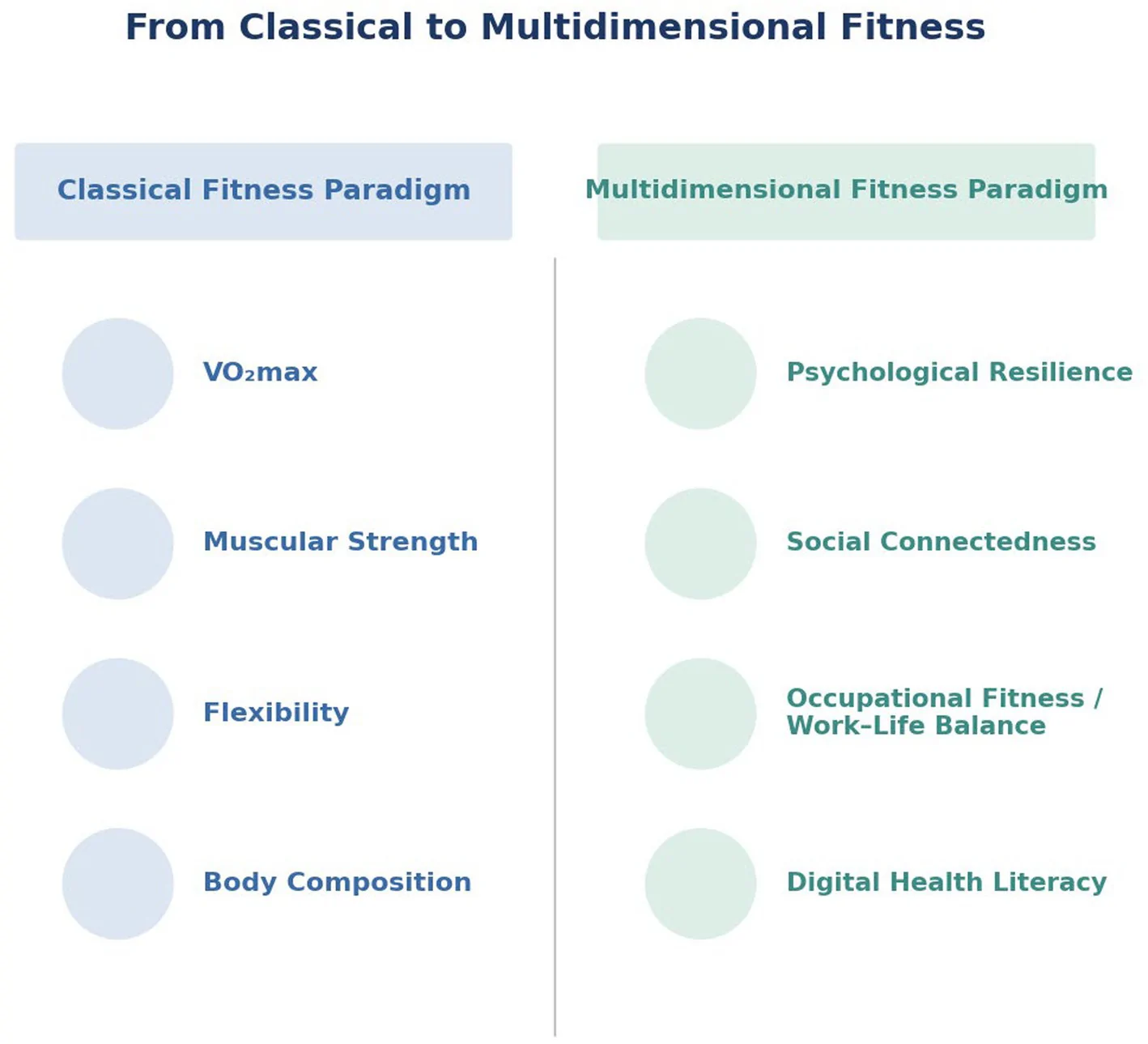
Transition from classical to multidimensional fitness paradigms. This figure contrasts the traditional physiological model of fitness—focused on VO₂max, muscular strength, flexibility, and body composition—with the proposed multidimensional paradigm that integrates psychological resilience, social connections, occupational balance, and digital health literacy as core determinants of holistic human functionality.

#### 1.3.2 Conceptual and methodological limitations

Despite these contributions, several critical limitations constrain the explanatory power and applicability of the classical paradigm in contemporary contexts (Figure 2):

- Psychological and emotional processes are marginalized. Traditional accounts rarely integrate constructs such as self-regulation, basic psychological needs, cognitive control, resilience, and emotion regulation—factors that are central to adherence, recovery, and performance sustainability (11–14).
- Social context is under-specified. Although social relationships strongly influence morbidity, mortality, health behaviors, and stress buffering (15, 16), fitness continues to be operationalized almost exclusively as an individual attribute, neglecting the structural and interactional determinants that shape opportunities for health (7, 17).
- Occupational realities are overlooked. Work demands, job control, reward structures, recovery opportunities, and burnout are powerful determinants of long-term health and functionality (18–21), yet they remain virtually absent in classical fitness frameworks.

**Figure 2:**
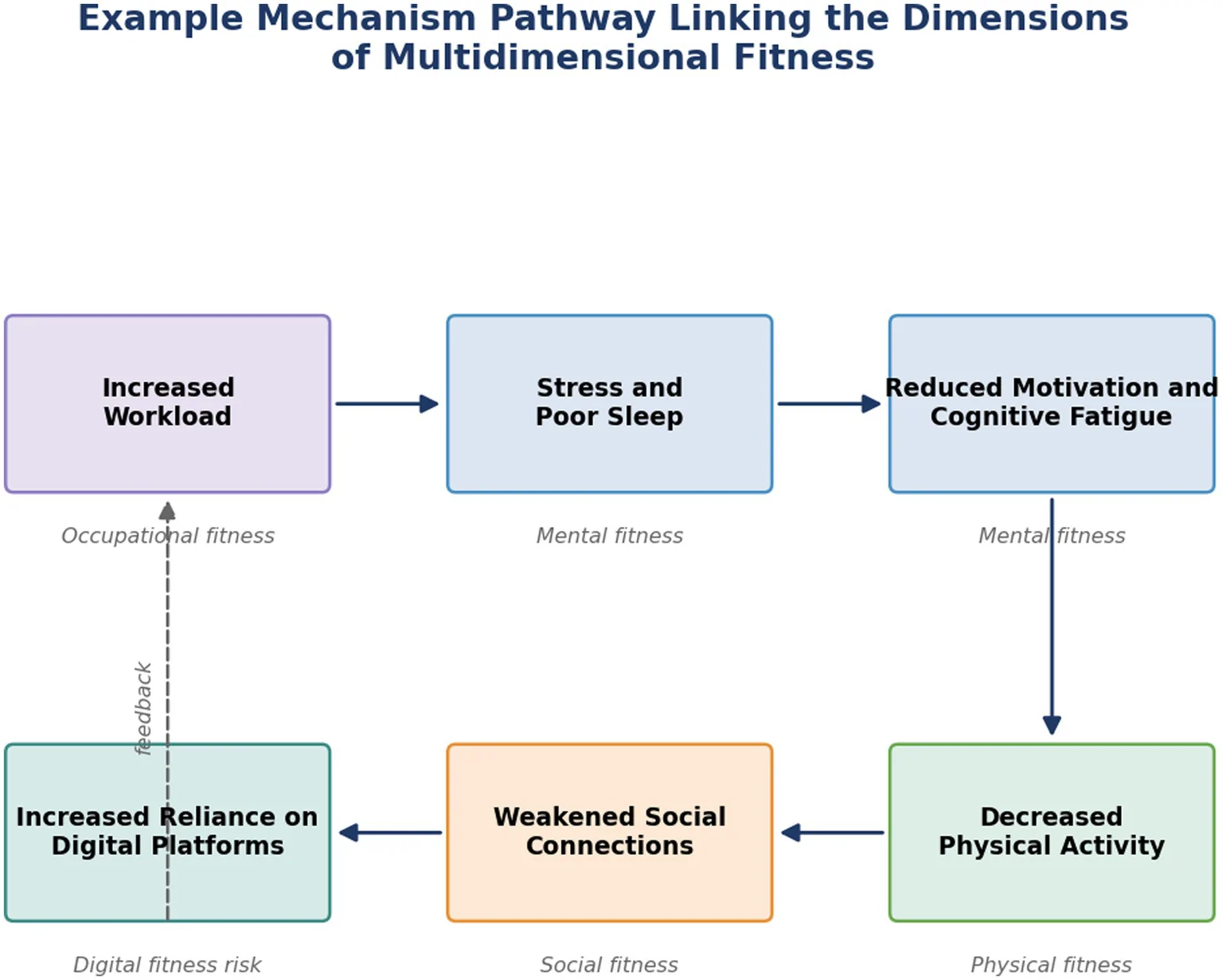
Interacting pathways linking occupational, mental, social, and digital fitness dimensions. This diagram illustrates how increased workload can trigger stress and poor sleep, leading to reduced motivation and cognitive fatigue that diminish physical activity. These changes progressively weaken social connections and heighten reliance on digital platforms, thereby revealing interdependent vulnerabilities across mental, social, and digital fitness dimensions within the multidimensional fitness framework.

Digital determinants are largely excluded. In the 21st century, ubiquitous devices, platforms, and algorithms mediate movement, attention, sleep, and social interaction. Wearables and AI-driven systems hold potential to augment fitness behaviors but also raise risks related to privacy, surveillance, and algorithmic inequities (9, 22, 23).

#### 1.3.3 Why this matters

These limitations are not trivial omissions—they highlight a structural misalignment between traditional fitness models and contemporary lived realities. Without incorporating psychological, social, occupational, and digital determinants, the concept of fitness risks being reduced to physiology alone, thereby failing to capture the complex biopsychosocial and technological forces that shape health, performance, and wellbeing in modern societies. Addressing these gaps is therefore essential to developing a multidimensional paradigm that maintains the scientific rigor of physiology while expanding its scope to reflect the full spectrum of determinants that enable sustainable human flourishing.

These omissions carry concrete costs, and those costs are unevenly distributed. In public health, surveillance systems and national guidelines that operationalize fitness as physiology and activity volume alone create a monitoring blind spot: population data capture moderate-to-vigorous activity minutes and, increasingly, sedentary time, but not social isolation, work strain, recovery deficit, or digital exclusion. Priorities and budgets follow what is measured, so programs are designed to move the indicators that surveillance reports; the plateau in global inactivity over two decades of sustained guideline promotion suggests the limits of that strategy (24, 25). The economic scale of the shortfall is substantial: physical inactivity alone was estimated to cost health-care systems approximately INT$53.8 billion in 2013 (26)—a figure that captures none of the productivity and care costs attributable to burnout, isolation, or recovery failure, which a broader framing would render visible and therefore actionable.

In fitness technology design, the physiological framing is encoded directly into products. Consumer wearables and applications are optimized around what classical models define as fitness—steps, heart rate, estimated VO₂max, energy expenditure—and consequently treat recovery, social connectedness, work demands, and the user’s own digital literacy as lying outside the design space rather than as legitimate targets. The predictable results are high non-adherence and early abandonment (9, 27); engagement mechanics that intensify self-surveillance among users already vulnerable to it (28); and default designs that presume discretionary time, disposable income, and confident device use, thereby widening rather than narrowing the digital divide (29). Naming digital and occupational capacities as constituents of fitness makes these design decisions explicit, auditable, and empirically testable rather than incidental.

In healthcare, exercise referral and lifestyle prescription built on physiological targets alone routinely fail for reasons the underlying model cannot represent. A patient whose binding constraint is rotating shift work, an absence of recovery opportunity, caregiving load, or the loss of a single supportive tie receives a dose prescription and is subsequently recorded as non-adherent—a misattribution of a structural constraint to individual motivation that then justifies further individual-level exhortation. Emerging referral models that target social connection directly illustrate both the promise of acting on these determinants and the current immaturity of the evidence for doing so (30).

The burden of this misalignment falls predictably: on shift and precarious workers, unpaid caregivers, older adults with contracting social networks, low-income and rural populations with limited device or broadband access, and people with disabilities for whom standard test batteries and application interfaces were never designed. For these groups a physiology-only definition of fitness does not merely omit relevant information; it systematically renders their principal constraints invisible to assessment and therefore ineligible for intervention, while attributing the resulting outcomes to personal choice.

Section 1.7 (Implementation in Practice: Examples and Use Cases) makes these implications concrete. It works through a lifestyle clinic that adds recovery, social-network, work-ability, and eHealth-literacy screening to standard intake and routes each finding to a distinct referral pathway; a knowledge-work firm that redesigns job demands and decision latitude instead of exhorting individual resilience; and a municipality that pairs built-environment change with device lending and digital-literacy provision in the neighbourhoods carrying the highest inactivity burden. In each case the decisive intervention is one that a physiology-only model would not have recognized as a fitness intervention at all.

### 1.4 Theoretical foundations for a multidimensional model

#### 1.4.1 The biopsychosocial tradition

A substantial body of research in behavioral medicine and social epidemiology demonstrates that health emerges through the interaction of biological processes, psychological states, and social environments. This tradition underscores that human functioning cannot be reduced to physiology alone. Transdisciplinary calls have further emphasized the integration of environmental and cultural determinants into physical activity promotion, reinforcing the need for a broader, systems-level view of fitness (8, 31).

#### 1.4.2 Behavior change science

Long-term adoption of fitness behaviors depends on the interplay of capability, opportunity, and motivation (COM-B framework) (32). Likewise, self-determination theory (11) highlights the necessity of fulfilling psychological needs for autonomy, competence, and relatedness to ensure sustained engagement. Programs that fail to explicitly address these levers often yield short-lived or unsustainable gains (14), highlighting the importance of embedding motivation science into fitness interventions.

#### 1.4.3 Cognitive control and resilience

Fitness also requires robust executive functions, such as shifting, updating, and inhibitory control, which support self-regulation, skill acquisition, and decision-making under stress (33, 34). In parallel, psychological resilience (35) and adaptive emotion regulation (13) act as moderators that determine whether stressors translate into constructive action or functional impairment. These processes are therefore central to sustaining performance and recovery across diverse contexts.

#### 1.4.4 Social integration and support

Meta-analytic evidence demonstrates that social connection predicts survival to a degree comparable with well-established biomedical risk factors (15). Moreover, social identities and networks shape norms, resource access, and habit formation, all of which directly influence movement patterns, recovery processes, and long-term health trajectories (16, 36). Thus, social fitness emerges as a determinant on par with physical activity itself, requiring explicit integration into any multidimensional model.

#### 1.4.5 Work design and recovery

Occupational frameworks such as the job demands–control model (18) and the effort–reward imbalance model (20) demonstrate how work structures shape health outcomes, particularly through chronic stress, fatigue, and burnout. Recovery science further emphasizes that opportunities for detachment and restoration are essential for sustained energy, productivity, and wellbeing (21, 37). Without addressing occupational contexts, fitness frameworks risk overlooking one of the most pervasive determinants of adult health.

#### 1.4.6 Digital health and ethics

In the digital era, technologies such as wearables, mobile applications, and AI-driven platforms have the capacity to scale personalized feedback and coaching. However, they simultaneously introduce risks concerning privacy, algorithmic bias, and governance. WHO guidance stresses the need for safety, equity, and human oversight in the use of AI for health (23), while ethical frameworks further highlight transparency and accountability (22). Given this landscape, digital literacy—the ability to critically evaluate and manage digital health information—must be considered a fundamental dimension of modern fitness (38).

#### 1.4.7 A multidimensional fitness paradigm

We propose that fitness be redefined as a dynamic capacity to mobilize and sustain physical, mental, social, occupational, and digital resources in order to meet the evolving demands of daily life and societal participation, in ways that are both ethical and sustainable across the lifespan. This paradigm highlights five interacting dimensions, which together provide a more comprehensive understanding of human functionality in contemporary contexts (Figure 3).

**Figure 3:**
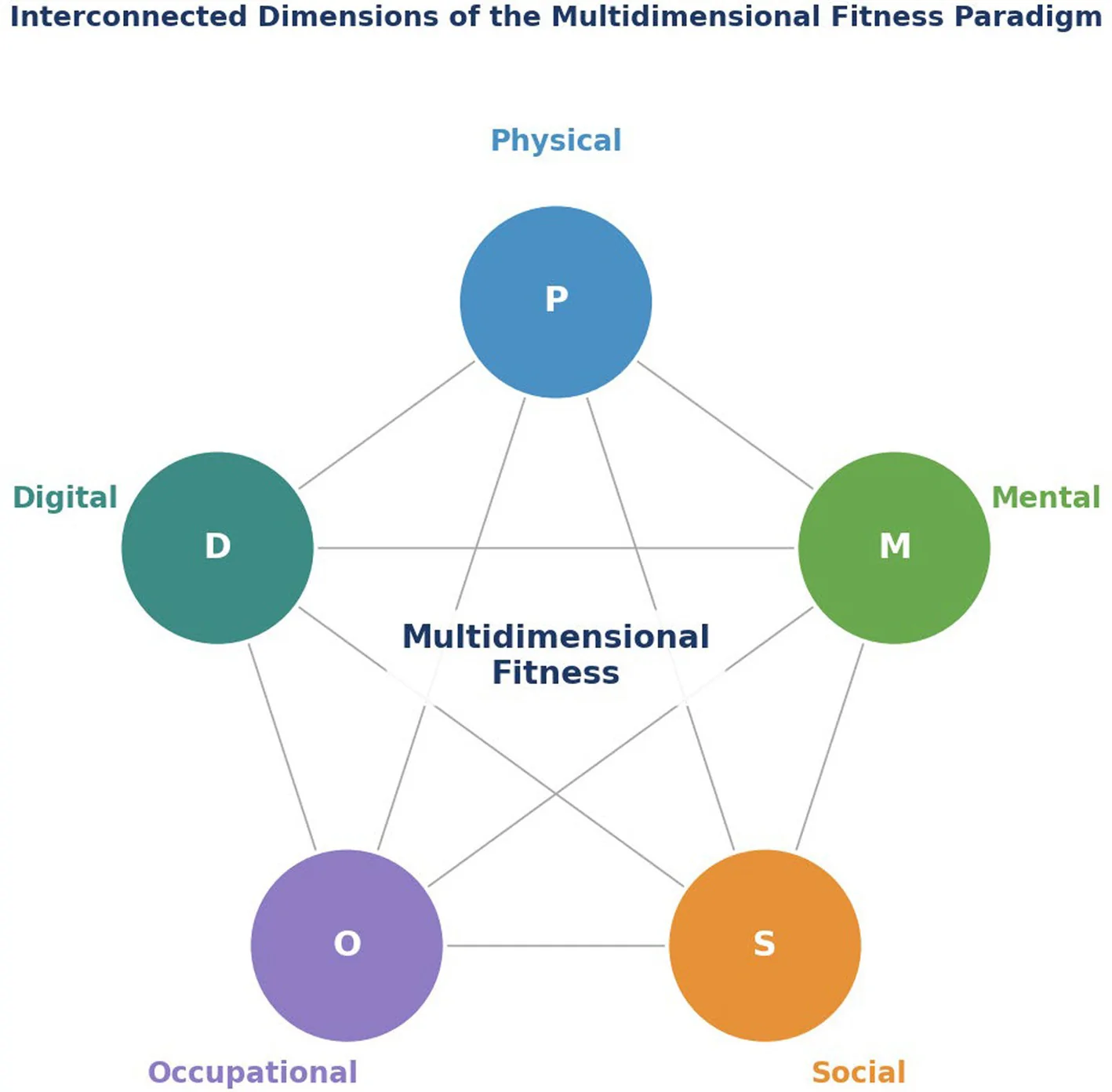
Interconnected dimensions of the multidimensional fitness paradigm. This schematic represents the five interdependent dimensions—physical, mental, social, occupational, and digital fitness—illustrating their bidirectional relationships. Each dimension dynamically influences and supports the others, forming an integrated biopsychosocial framework essential for sustaining holistic health, performance, and well-being across the lifespan.

The selection of these five dimensions—and no others—was not arbitrary. Each dimension satisfies three conditions simultaneously: (a) theoretical salience, i.e., an independent, well-documented evidence base linking the underlying capacities to health and functional outcomes (physical: consensus exercise-science guidelines; mental: executive-function, emotion-regulation, and resilience research; social: meta-analytic evidence linking social connection to mortality; occupational: the demand–control and effort–reward traditions; digital: eHealth literacy and digital-divide research); (b) measurement maturity, i.e., availability of validated individual-level instruments; and (c) intervenability, i.e., documented responsiveness to structured intervention. The formal inclusion criteria, and the constructs excluded by them, are specified in the Conceptual Boundaries subsection below (Figure 4).

1. Physical fitness. Defined as the energetic and efficient execution of daily activities and the capacity to respond protectively to physical stressors. It encompasses cardiorespiratory endurance, neuromuscular strength, motor control, and body composition (1, 3).
2. Mental fitness. Encompasses cognitive and affective capacities such as executive control, attentional regulation, emotion regulation, and resilience, which enable adaptive functioning under uncertainty and stress (12, 13, 35).
3. Social fitness. Refers to the ability to cultivate, mobilize, and sustain supportive, diverse, and reciprocal social ties, and to effectively participate in community structures—determinants strongly linked to morbidity, mortality, and wellbeing (15, 16).
4. Occupational fitness. Denotes sustainable performance and wellbeing in work contexts, grounded in healthy job design, recovery practices, skills for focus and collaboration, and the alignment of demands with available resources (18, 20, 21).
5. Digital fitness. Refers to the competent, ethical, and health-promoting use of digital tools—such as wearables, platforms, and AI-driven coaches—combined with digital literacy skills (e.g., information appraisal, privacy self-management) to mitigate risks and maximize benefits (9, 23, 38).

**Figure 4:**
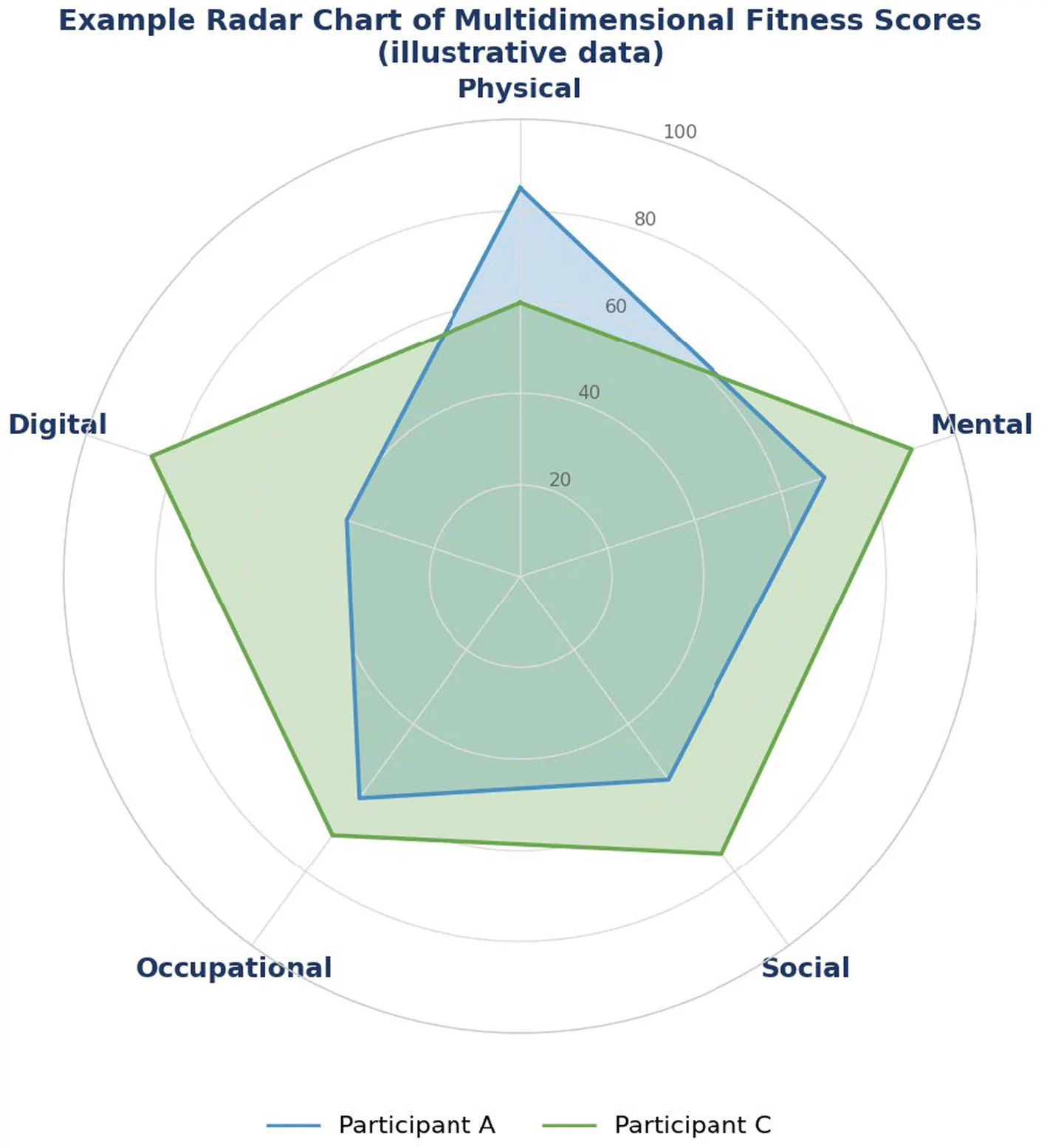
Example of multidimensional fitness profiling using radar visualization. This figure depicts individual differences in fitness across five dimensions—physical, mental, social, occupational, and digital. Participant A and Participant C exhibit distinct multidimensional profiles, illustrating how composite fitness assessment can identify relative strengths and vulnerabilities across interacting biopsychosocial and digital capacities.

Importantly, these dimensions are not siloed. Instead, they operate interdependently: social norms and workplace culture influence opportunities for movement and recovery; mental fitness supports motivation, adherence, and risk management; and digital tools can either augment or erode capacities across all dimensions. This systemic integration underscores the value of a multidimensional paradigm that transcends classical physiological models and aligns more closely with real-world determinants of health, performance, and wellbeing.

#### 1.4.8 Conceptual boundaries: what qualifies as a dimension of fitness—and what does not

To prevent the framework from becoming an unbounded relabeling of biopsychosocial determinants, we specify four conceptual criteria that a construct must jointly satisfy to qualify as a dimension of fitness: (a) it must denote a capacity of the person—an ability that can be mobilized to meet task and environmental demands—rather than a feature of the environment itself; (b) it must be trainable, that is, modifiable through structured practice or intervention; (c) it must be measurable at the individual level with validated instruments; and (d) it must demonstrably predict functional performance and health outcomes. Constructs that fail any criterion are treated as determinants, moderators, or outcomes of fitness—not as fitness dimensions.

This capacity-based definition distinguishes multidimensional fitness from adjacent frameworks. The biopsychosocial and social-ecological models describe determinants and levels of influence on health; multidimensional fitness, by contrast, specifies trainable capacities of persons. Wellbeing and quality-of-life frameworks emphasize subjective, evaluative states—outcomes—whereas fitness denotes the functional capacities that help produce such outcomes. Health is the broader end-state to which fitness contributes. In short, fitness stands to functioning as capability stands to achievement: it is a modifiable, assessable capacity, not a determinant, a context, or an outcome.

On these criteria, occupational and digital fitness are retained as dimensions because each denotes an individual capacity exercised within a context, not the context itself. Occupational fitness refers to trainable capacities such as recovery skills, boundary management, workload self-regulation, and focused collaboration under demand; job design, decision latitude, and reward structures are treated as contextual determinants that enable or constrain this capacity, and they enter the framework as moderators rather than as constituents. Analogously, digital fitness refers to competencies such as eHealth literacy, critical information appraisal, privacy self-management, and intentional device use; infrastructure, platform design, and the digital divide are contextual determinants rather than components of the construct.

Applying the same criteria, several candidate constructs were deliberately excluded. Nutrition, sleep, and socioeconomic position operate as cross-cutting determinants or resources that influence several dimensions simultaneously rather than as distinct trainable capacities; “financial fitness” and “spiritual fitness” currently lack sufficiently validated, cross-culturally robust measurement models; and environmental quality is a property of settings rather than of persons. These boundaries preserve the discriminant validity of the framework while acknowledging that the excluded constructs remain important covariates in empirical work.

### 1.5 Dimension by dimension: concepts, mechanisms, measures, and caveats

#### 1.5.1 Physical fitness

##### 1.5.1.1 Concept and mechanisms

Physical fitness reflects integrated adaptations across cardiovascular, respiratory, neuromuscular, metabolic, and motor systems. Mechanistic pathways include enhanced endothelial function, mitochondrial biogenesis, neuromuscular coordination, and anti-inflammatory effects.

##### 1.5.1.2 Candidate measures

VO₂max or validated submaximal proxies; functional field tests (e.g., 6-min walk, shuttle run); strength and power tests (1RM, isometric dynamometry, vertical jump); muscular endurance (sit-to-stand, plank time); mobility and balance (timed up-and-go, gait speed, stance tasks); and body composition (DXA, anthropometry). Ecological metrics from wearables (e.g., step counts, active minutes, gait variability) hold promise, though validity varies across devices and populations (9).

##### 1.5.1.3 Interventions

Multicomponent programs combining aerobic, resistance, and neuromotor training remain the gold standard (1, 6). WHO (3) emphasizes the principle of “move more, sit less,” with resistance training across all age groups.

Caveats. Overemphasis on performance can obscure motivational and psychological barriers (39, 40). Sedentary exposure interacts with physical activity dose: high levels of moderate-to-vigorous activity may offset sitting risks, but inactive individuals remain particularly vulnerable (5). Moreover, equity concerns persist in contexts lacking safe public spaces and accessible facilities (8).

#### 1.5.2 Mental fitness

##### 1.5.2.1 Concept and mechanisms

Mental fitness encompasses executive functions (updating, shifting, and inhibition), emotion regulation, stress appraisal, and resilience—capacities central to attention, learning, and behavior under stress (12, 13, 34, 35). Exercise itself enhances cognition and mood through neurotrophic, vascular, and psychosocial mechanisms (41).

##### 1.5.2.2 Candidate measures

Executive tasks (e.g., n-back, Stroop, task switching); validated questionnaires of emotion regulation (ERQ) and resilience (CD-RISC); indices of perceived stress, sleep quality, and cognitive fatigue; and ecological momentary assessment for affect and self-regulation.

##### 1.5.2.3 Interventions

Self-determination theory (SDT)–informed coaching that fosters autonomy, competence, and relatedness (14); cognitive-behavioral strategies (implementation intentions, planning, self-monitoring); mindfulness and reappraisal training; structured recovery and sleep protocols; and graded exposure to challenge.

##### 1.5.2.4 Caveats

Assessments are sensitive to learning effects and context; cross-cultural validity must be confirmed. Popular “quick fixes” risk trivializing complexity, as durable improvements often require structural changes in environments, not just skill training.

#### 1.5.3 Social fitness

##### 1.5.3.1 Concept and mechanisms

Social fitness reflects both the quantity and quality of relational ties—family, peers, coworkers, community—as well as the skills required to build and sustain them. Mechanisms include social control of health behaviors, emotional support, informational and instrumental aid, and identity-based motivation (15, 16, 36).

##### 1.5.3.2 Candidate measures

Social network indices (Berkman–Syme, Lubben); perceived support surveys (MOS-SS); group participation and prosocial activity; loneliness scales; and digital sociality metrics (with privacy safeguards).

##### 1.5.3.3 Interventions

Group-based physical activity; community and urban design fostering walkability and incidental encounters (8); peer mentorship and identity-based health messaging; workplace social architecture that values psychological safety and active breaks.

##### 1.5.3.4 Caveats

Social capital is unequally distributed, and interventions risk reinforcing exclusion where access barriers persist (17). Not all ties are health-promoting (e.g., norms discouraging activity). Ethical considerations in mapping social networks are essential.

#### 1.5.4 Occupational fitness

##### 1.5.4.1 Concept and mechanisms

Occupational fitness denotes the capacity to perform, learn, and thrive in work environments. Mechanisms include the balance of demands and resources, job control, reward structures, and recovery opportunities (18, 20, 21). Chronic imbalance can lead to fatigue, presenteeism, and burnout (19).

##### 1.5.4.2 Candidate measures

Work Ability Index; Job Content Questionnaire; effort–reward imbalance scales; burnout inventories; Recovery Experience Questionnaire; workload assessments; and metrics of focus versus interruptions.

##### 1.5.4.3 Interventions

Job redesign to increase autonomy and fairness; “right to disconnect” norms; workload leveling; leadership training in sustainable performance; movement micro-breaks; and sleep-supportive scheduling.

##### 1.5.4.4 Caveats

Placing responsibility on individuals (e.g., resilience workshops) without systemic reform is ineffective and ethically problematic. Interventions should be co-designed with employees, with sensitivity to equity and caregiving demands.

#### 1.5.5 Digital fitness

The concept of digital fitness represents an emergent multidimensional construct situated at the intersection of technology, health behavior, and data ethics. It involves the health-promoting use of wearables, mobile applications, digital platforms, and AI-based coaching systems—combined with individual competencies to evaluate information quality, safeguard privacy, and navigate algorithmic environments (38). Conceptually, digital fitness extends beyond physical activity tracking to encompass cognitive and ethical literacies that enable sustainable engagement with digital ecosystems. Mechanistically, its operation depends on dynamic feedback loops, habit scaffolding, and social reinforcement processes that mediate user adherence and motivation; however, these same mechanisms may also generate risks such as surveillance fatigue, data misuse, and inequitable access to digital infrastructures (9, 23, 29).

A clarification is warranted at this point, because digital fitness as described above spans two analytically distinct objects: the competencies of the person, and the properties of the technologies that person uses. Only the first constitutes a dimension of fitness under the criteria specified in Section 1.3.8 (Conceptual Boundaries). Digital fitness proper denotes person-level, trainable, individually measurable capacities: eHealth literacy and critical appraisal of health information; privacy and security self-management; the ability to calibrate trust in algorithmic recommendations; intentional, boundary-managed device use; and the capacity to convert digital feedback into sustained behavior. Platform and device attributes—interface usability, sensor validity and interoperability, algorithmic transparency and contestability, default notification and data-sharing settings, accessibility provision, subscription cost, and the availability of human-in-the-loop support—are properties of artifacts rather than of persons. They are neither trainable within the individual nor measurable as individual attributes, and they therefore fail criteria (a) and (c).

These technology attributes are nonetheless indispensable to the framework, and they enter it in precisely the position that job design occupies for occupational fitness: as contextual determinants and moderators, not as constituents of the person’s capacity. Three operational consequences follow. First, the digital fitness dimension score is computed only from person-level indicators (e.g., eHEALS, privacy-literacy items, indices of intentional use); platform attributes are recorded on a parallel context register and are never aggregated into that score. Second, the substantive prediction is interactional rather than additive: the health return on individual digital capacity is expected to depend on the design environment, such that high capacity is partly wasted on opaque or extractive platforms, while transparent, well-designed, low-cost tools partially compensate for low capacity. This person × platform interaction is directly testable and would be obscured if both were folded into a single score. Third, the two objects imply different intervention levers: individual capacity is addressed through literacy training and coaching, whereas design is addressed through procurement standards, transparency requirements, and technology governance (Section 1.10, Policy Implications and Recommendations)—which is why the framework’s digital recommendations are directed at regulators, employers, and system purchasers as well as at individuals. This separation is consistent with established technology-acceptance research, which distinguishes user-level expectancies from facilitating conditions supplied by the environment (42), and with AI governance frameworks that assign transparency and contestability obligations to system providers rather than to users (22, 23).

Despite its growing relevance, digital fitness research faces profound methodological challenges. Current measurement frameworks rely heavily on trace data obtained from wearables and health-tracking applications, raising concerns about data validity, standardization, and ethical governance. The intertwined nature of online and offline behaviors complicates the isolation of causal mechanisms and challenges the ecological validity of observed effects (43). Likewise, cross-platform inconsistencies in sensor calibration and data fusion—as illustrated in the Digital Fitness Connector architecture proposed by Gupta and Jilla (44)—highlight persistent reliability and interoperability issues (44).

Qualitative investigations further reveal the interpretive and experiential complexity of digital fitness. Depper and Howe (28) demonstrated that health and fitness apps can reshape adolescents’ embodied self-regulation and surveillance perceptions, complicating the distinction between subjective experience and empirical observation. Similarly, Gulati et al. (45) reported that young adult users express ambivalence regarding device accuracy, motivational decline, and digital dependence, which in turn undermines long-term adherence and data continuity (45). At the conceptual level, definitional ambiguity persists: Kossowski, Lenz, Heumüller, and Richter (46) conceptualized digital fitness as the target state of digital transformation—encompassing digital expertise, innovation adoption, and adaptive capacity—yet acknowledged the absence of robust, measurable indicators (46). Collectively, these findings underscore ongoing methodological fragmentation and the need for transdisciplinary operationalization strategies (28, 43–46).

To advance empirical precision, candidate measures for digital fitness assessment include eHealth literacy (eHEALS), privacy and security literacy, device engagement quality (beyond mere frequency), AI fairness and transparency audits, adoption and retention metrics, and net behavioral change outcomes. Effective interventions should integrate evidence-based wearable strategies—such as goal-setting, self-monitoring, prompts, and social comparison—with transparent AI coaching systems that preserve autonomy and privacy. Moreover, platform hygiene practices (notification control, data minimization), human-in-the-loop safeguards, and equitable access initiatives are indispensable to ensuring sustainable participation.

Applying this distinction to the measures just listed clarifies their role in the model. eHealth literacy, privacy and security literacy, and engagement quality index the person and contribute to the dimension score. AI-coach transparency, opt-out availability, data-minimization compliance, and the provision of accessibility features index the platform and are recorded as contextual moderators. Adoption and retention metrics are joint products of person and platform and are best modeled as outcomes rather than as inputs to either.

Nevertheless, empirical evidence indicates that effect sizes for digital interventions remain highly variable. Benefits often diminish without human facilitation or context-sensitive personalization. Consequently, the long-term success of digital fitness initiatives depends on ethical guardrails, inclusivity, accessibility, and the sustained integration of human oversight within AI-driven environments (23).

#### 1.5.6 Uneven maturity of the evidence base

The evidential status of the five dimensions is deliberately not treated as equivalent. Physical fitness rests on established evidence: decades of experimental and epidemiological research codified in consensus guidelines (1, 3). The occupational constructs (demand-control, effort-reward imbalance, recovery) are likewise supported by mature longitudinal evidence, although their integration into a fitness framework is novel. Mental and social fitness are supported by robust evidence for their component constructs (executive function, emotion regulation, resilience, social connection) but by emerging evidence as trainable, integrated fitness dimensions. Digital fitness remains largely a conceptual proposition: its constituent literacies are measurable, but the construct is heterogeneous and its predictive validity is untested. Table 1 summarizes this gradient, and the strength of claims made throughout the manuscript—and of the policy recommendations below—is calibrated to it.

**Table 1:** The five proposed fitness dimensions: working definitions, key mechanisms, candidate measures, evidence maturity, and principal limitations.

Dimension	Working definition	Key mechanisms	Candidate measures	Evidence maturity	Principal limitations
Physical	Energetic, efficient execution of daily activities; protective response to physical stressors	Cardiorespiratory, neuromuscular, metabolic, and motor adaptations	VO2max or submaximal proxies; grip strength; sit-to-stand; gait speed; MVPA minutes; device-measured sedentary time	Established	Performance focus may obscure motivational barriers; wearable validity varies across devices and populations
Mental	Cognitive-affective capacities enabling adaptive functioning under uncertainty and stress	Executive control; emotion regulation; stress appraisal; resilience	n-back, Stroop, task switching; CD-RISC; ERQ; perceived stress; sleep efficiency	Emerging (component constructs established)	Practice and context effects; cross-cultural validity requires confirmation
Social	Ability to build, mobilize, and sustain supportive, reciprocal ties and participate in community structures	Social support; social control of health behaviors; identity-based motivation	Berkman-Syme index; LSNS-6; MOS-SS; loneliness scales; participation indices	Emerging (component constructs established)	Unequal social capital; not all ties are health-promoting; ethical issues in network mapping
Occupational	Sustainable performance and wellbeing capacities in work contexts	Demand-resource balance; job control; reward; recovery and detachment	WAI; JCQ; ERI; REQ; burnout inventories	Established constructs; novel as a fitness dimension	Individual-level focus risks displacing systemic workplace reform
Digital	Competent, ethical, health-promoting use of digital tools plus protective literacies	Feedback loops; habit scaffolding; social reinforcement	eHEALS; privacy/security literacy; engagement quality; adherence to device-based goals	Largely conceptual; heterogeneous	Definitional ambiguity; trace-data validity; digital divide; predictive validity untested

### 1.6 Cross-cutting issues

#### 1.6.1 Equity and inclusion

Global surveillance data reveal persistent—and in some cases widening—inequities in physical activity participation, particularly by income and gender (24, 25). These inequities are compounded by a digital divide in access, literacy, and cultural relevance, which determines who can benefit from technology-enabled fitness interventions (29). Accordingly, advancing a multidimensional fitness paradigm must be pursued as an equity project, emphasizing universal access to safe spaces for movement, culturally attuned program design, affordable tools, and inclusive participation across socioeconomic and demographic groups. Without explicit equity safeguards, the paradigm risks reinforcing existing health disparities (Figure 5).

**Figure 5:**
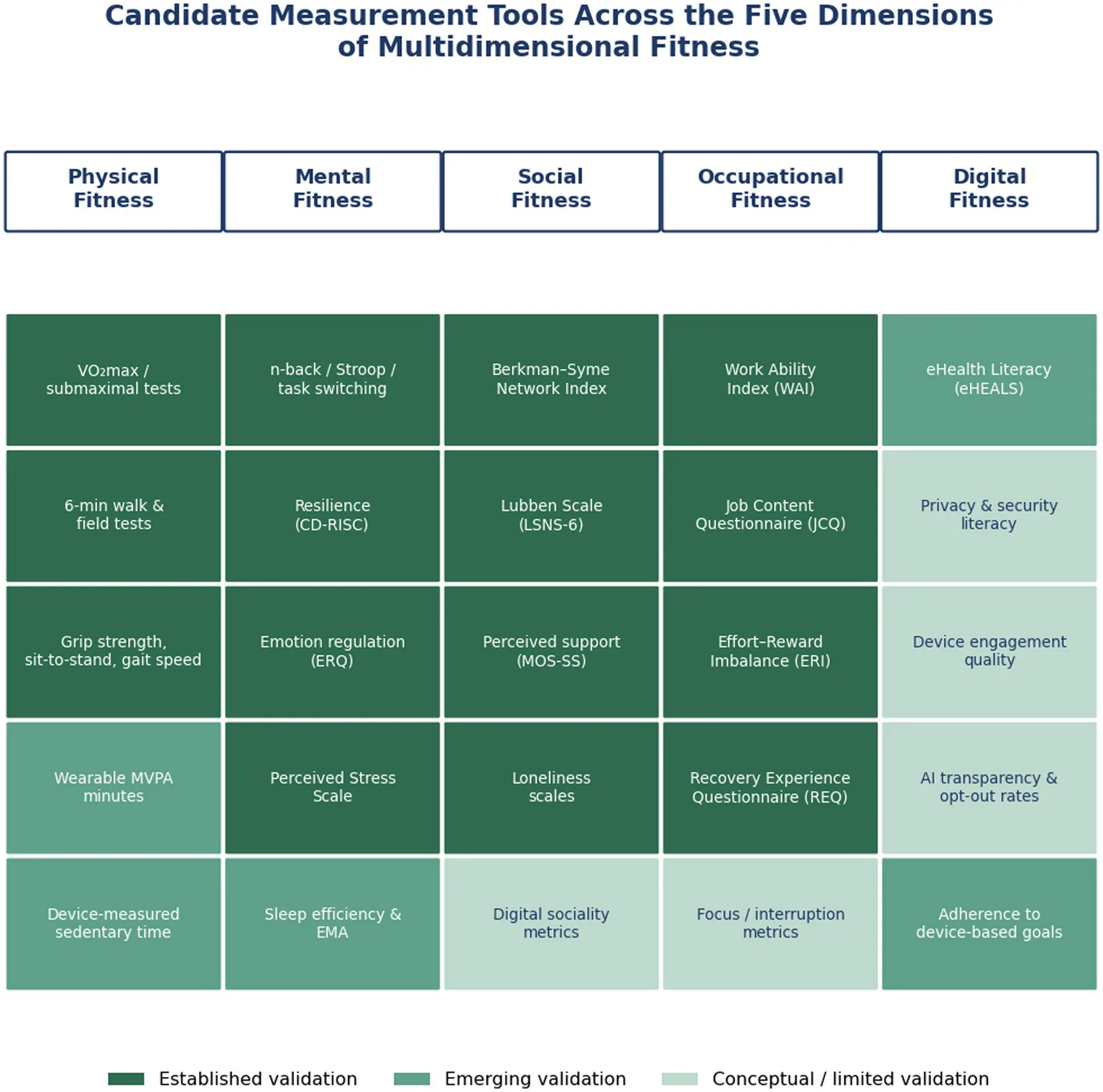
Candidate measurement framework across the five dimensions of multidimensional fitness. This figure summarizes validated and emerging assessment tools for physical, mental, social, occupational, and digital fitness dimensions. It integrates laboratory tests, self-report questionnaires, ecological sensor data, and digital metrics—ranging from VO₂max and resilience scales to eHealth literacy and device adherence—illustrating a multimethod approach for operationalizing and validating holistic fitness in research and practice. Color coding denotes evidence maturity: established, emerging, or conceptual/limited validation.

#### 1.6.2 Ethics, privacy, and governance

The rapid integration of AI-enabled fitness technologies raises profound concerns regarding data ownership, consent, transparency, and algorithmic bias. WHO guidance recommends principles of human oversight, safety, accountability, equity, and sustainability (23), while independent AI ethics frameworks reinforce transparency and procedural protections (22). Organizations should therefore adopt privacy-by-design architectures, conduct algorithmic impact assessments, and ensure that fitness technologies are never used for punitive or coercive purposes. Ethical governance is not a peripheral issue but a prerequisite for public trust and sustainable implementation.

#### 1.6.3 Culture and environment

Fitness cannot be disentangled from the built environment and cultural context in which individuals live. Walkability, green space, and public transit infrastructure shape opportunities for movement, while cultural norms influence motivation and participation (8). Importantly, programs must be context-sensitive: interventions that are effective in dense urban environments may be ineffective—or even counterproductive—in rural or resource-limited settings. Embedding cultural and environmental specificity into design is therefore central to ensuring relevance, scalability, and adoption.

#### 1.6.4 Lifespan perspective

Fitness capacities evolve and decline across the lifespan. Early-life motor competence predicts later activity trajectories, while midlife work and caregiving responsibilities shape recovery and stress exposure. In older adulthood, priorities shift toward balance, muscle power, and social connectedness as determinants of independence and wellbeing. Digital tools also require age-appropriate accessibility, ensuring that both younger and older populations can benefit. Adopting a lifespan perspective thus ensures that multidimensional fitness frameworks remain responsive to developmental windows, age-specific needs, and the changing determinants of health across the human life course.

### 1.7 Operationalizing the multidimensional paradigm

#### 1.7.1 Construct specification

##### 1.7.1.1 Latent structure

Multidimensional fitness can be conceptualized as a higher-order latent construct reflected by five first-order dimensions: physical, mental, social, occupational, and digital. While each dimension represents a distinct factor, correlated residuals are expected because determinants such as sleep, recovery, and stress influence multiple dimensions simultaneously.

##### 1.7.1.2 Measurement model

To capture this multidimensionality, measurement must integrate performance-based assessments, validated questionnaires, ecological sensor data, and administrative indicators. A multi-method, multi-informant design with harmonized metadata is preferable, as it enhances construct validity and facilitates comparability across studies and contexts.

#### 1.7.2 Candidate Indicator set (illustrative)

An initial indicator framework could draw from validated tools and ecologically valid measures across the five dimensions.

Instruments were mapped to dimensions using three explicit criteria: (a) construct correspondence—each instrument operationalizes a mechanism specified for its dimension (e.g., the CD-RISC captures resilience within mental fitness; the LSNS-6 captures network structure within social fitness; the WAI captures perceived work capacity within occupational fitness; the eHEALS captures eHealth literacy within digital fitness); (b) psychometric evidence—published reliability and validity data, ideally across languages and age groups; and (c) feasibility—brief administration time, low cost, and availability of validated translations. Where several instruments met these criteria, the most widely validated and freely available option was preferred. Figure 5 mirrors this allocation.

- Physical: VO₂max (laboratory or submaximal proxies), grip strength, sit-to-stand performance, gait speed, balance (e.g., eyes-closed stance), weekly moderate-to-vigorous physical activity (MVPA) minutes, and device-measured sedentary time.
- Mental: n-back accuracy, task-switching cost, Stroop interference, resilience (CD-RISC), emotion regulation (ERQ: reappraisal/suppression), perceived stress, and sleep efficiency.
- Social: Berkman–Syme or Lubben Social Network scores, MOS-SS subscales, weekly prosocial engagement, reported loneliness, and group activity participation.
- Occupational: Work Ability Index, Job Content Questionnaire (demands/control/support), effort–reward imbalance ratio, Recovery Experience Questionnaire, burnout indices, and aggregated focus/interruptions per hour (with privacy protections).
- Digital: eHealth literacy (eHEALS), privacy and security literacy, AI-coach transparency and opt-out rates, adherence to wearable-based goals, data minimization compliance, and usage of accessibility features.

#### 1.7.3 Why these indicators, what they cost, and how much is enough

The set above is illustrative rather than prescriptive, and it is deliberately weighted toward instruments that are free, brief, widely translated, and already embedded in existing cohorts—properties that matter more for a framework intended to be adopted than marginal gains in measurement precision. Each choice nevertheless embodies a trade-off that implementers should make deliberately rather than inherit, and the paragraphs below make those trade-offs explicit.

##### 1.7.3.1 Physical

Directly measured VO₂max remains the criterion standard and carries the strongest outcome evidence, but it requires a laboratory, trained personnel, medical screening, and roughly an hour per participant, which makes it infeasible for most intake settings and for all population surveillance. Validated submaximal and field alternatives (step tests, six-minute walk, shuttle run) reduce cost by an order of magnitude at the price of wider individual prediction error; they are generally adequate for detecting within-person change even where they are inadequate for individual classification against normative cut-points. Grip strength illustrates the opposite trade-off: a single two-minute measure with documented prognostic value for cardiovascular events and all-cause mortality across high-, middle-, and low-income countries (47), yet a poor proxy for lower-limb function—which is why sit-to-stand and gait speed are retained alongside it rather than replaced by it. Device-measured activity and sedentary time are less biased than self-report but introduce wear-compliance loss, device heterogeneity, and non-random missingness; where devices cannot be issued to everyone, a validated short questionnaire administered to all is preferable to device data from a self-selected subsample.

##### 1.7.3.2 Mental

Performance-based executive tasks (n-back, Stroop, task switching) have the strongest construct validity and are the least practical component of the battery: they require standardized administration, are sensitive to practice effects on repeated testing, lack population norms in many languages, and correspond only modestly with everyday self-regulation. Where the purpose is screening or change monitoring rather than cognitive phenotyping, validated self-report instruments (CD-RISC, ERQ, perceived stress) deliver most of the actionable signal at a fraction of the burden; the performance tasks are better reserved for research contexts and for cases in which objectively verified cognitive change is the specific question being asked.

##### 1.7.3.3 Social

Full social network interviews are the most informative and the least deployable option. The LSNS-6 provides a six-item structural index with established cut-points for network vulnerability, and the three-item UCLA short form provides a validated loneliness measure in under a minute (48). Administered together they take approximately 2 min and separate structural isolation from perceived isolation—the distinction that determines whether the appropriate response is network building or relational quality. Perceived-support scales add depth but overlap substantially with both and are therefore assigned to the extended rather than the core tier.

##### 1.7.3.4 Occupational

The full Work Ability Index is comprehensive but long; the single-item work ability score performs well as a screen and predicts disability and early exit from working life, making it the defensible core-tier choice, with the Job Content Questionnaire, effort–reward imbalance ratio, and recovery and burnout inventories reserved for settings in which the results will actually inform job redesign (49). Passive workplace telemetry (focus blocks, interruptions per hour) is analytically attractive and ethically hazardous: it should be collected in aggregate only, never at the individual level, and only where employee representatives have agreed to its use in advance.

##### 1.7.3.5 Digital

eHEALS remains the most widely validated eHealth literacy instrument, but it predates conversational AI and algorithmic recommendation and therefore under-covers appraisal of AI-generated health advice and calibrated trust in automated coaching. Implementers should expect to supplement it, and the development and validation of an AI-era digital fitness measure is among the clearest measurement gaps in the framework (Section 1.9, A 10-Point Research Agenda).

##### 1.7.3.6 Matching the battery to the decision

The operative question in practice is not which measures are best in the abstract, but which decision the measurement will inform. A battery that exceeds the decision it serves imposes burden without benefit and depresses completion, while a battery that falls short of it generates data no one can act on. We therefore propose a tiered structure (Table 2). Tier 1 is a core screen of approximately 20–25 min requiring no laboratory and no proprietary instruments, sufficient to generate a five-dimension profile, to flag which dimensions warrant attention, and to support repeated measurement over time. Tier 2 adds dimension-specific depth and is triggered selectively by Tier 1 results—a screen-and-confirm logic that concentrates burden on those participants for whom additional information will change management. Tier 3 comprises laboratory and research-grade assessment appropriate to validation and mechanistic studies rather than to routine practice.

**Table 2:** Proposed tiered indicator structure: Core screen, triggered extension, and research-grade assessment, with the principal trade-off governing each dimension.

Dimension	Tier 1 — Core screen (≈20–25 min, no laboratory)	Tier 2 — extended (triggered by Tier 1)	Tier 3 — research/laboratory	Principal trade-off
Physical	Grip strength; 30-s sit-to-stand; 4-m gait speed; submaximal step test; short activity/sedentary questionnaire	7-day accelerometry; six-minute walk or shuttle run; balance battery; body composition	Direct VO₂max; 1RM or isometric dynamometry; DXA	Precision and outcome-evidence strength versus cost, equipment, screening burden, and clinical risk
Mental	Perceived stress; CD-RISC short form; ERQ; single-item sleep quality	Full CD-RISC; sleep diary or actigraphy-derived efficiency; brief computerized executive screen	n-back, Stroop, task-switching with standardized administration and norms	Construct validity of performance tasks versus practice effects, language norms, and administration burden
Social	LSNS-6; three-item UCLA loneliness scale; single item on group participation	MOS-SS subscales; prosocial engagement log; network diversity items	Full ego-network interview; ecological momentary assessment of interaction	Depth of relational information versus interview time and respondent fatigue
Occupational	Single-item work ability score; brief demand–control and recovery items	Full Work Ability Index; Job Content Questionnaire; effort–reward imbalance ratio; Recovery Experience Questionnaire; burnout inventory	Aggregate, consented workload telemetry; diary studies of detachment and recovery	Diagnostic value for job redesign versus survey length and, for telemetry, serious privacy and power asymmetries
Digital	eHEALS; two privacy self-management items; intentional-use item	Privacy and security literacy scale; engagement-quality metrics; AI-advice appraisal items	Platform audit, algorithmic impact assessment, and validated AI-era digital literacy instrument (to be developed)	Availability of validated instruments versus rapid technological change and the person–platform distinction (Section 1.4.5, Digital Fitness)

##### 1.7.3.7 Context-specific prioritization

In clinical and lifestyle-clinic intake, where consultation time is the binding constraint and the output is a treatment plan, priority belongs to indicators that are actionable within the clinic’s referral repertoire and responsive to intervention: a submaximal cardiorespiratory estimate, grip strength and sit-to-stand, a brief stress or resilience screen, LSNS-6 with the three-item loneliness scale, the single-item work ability score, and eHEALS—together achievable within a single visit. Where a service cannot accommodate all five dimensions, the ordering principle is actionability: a dimension should be measured only where a referral pathway exists for a poor result, since screening without a response pathway generates burden, and occasionally harm, without benefit. In workplace programs, occupational and recovery indicators carry the highest information value and must be collected, analyzed, and reported in aggregate only, with physical and digital components offered on an opt-in basis and never linked to employment decisions. In population surveillance, only brief self-report and device-based indicators are viable at scale, and the priority is comparability across waves rather than depth within them. In validation research the full battery is warranted, but planned-missingness designs allow the complete indicator set to be estimated at sample level without administering it in full to any single participant.

##### 1.7.3.8 Status of this proposal and future work

We emphasize that the tiering presented here is a proposal to be tested, not a validated protocol. The composition of the core tier, the cut-points that trigger extended assessment, the incremental predictive value contributed by each added indicator, and the acceptability, completion, and cost of each configuration in real settings are all empirical questions that this review cannot settle. Establishing a minimum core set is properly the work of a formal consensus process—a Delphi exercise involving clinicians, employers, community organizations and, critically, the people who will be assessed—conducted according to established methodology for core outcome set development (50) and followed by feasibility and burden testing in each target context. This is specified as a priority deliverable of the research agenda in Section 1.9 (A 10-Point Research Agenda).

#### 1.7.4 Scoring, aggregation, and reporting

Indicators should be normalized to z-scores within age, sex, and context strata. Dimension scores can then be computed as weighted composites, with weights empirically derived from factor analytic techniques. Reporting should include:

1. Absolute scores with confidence intervals;
2. Change over time (longitudinal sensitivity);
3. Equity-sensitive breakdowns (e.g., socioeconomic status, gender).

At the same time, reporting must be careful to avoid stigmatization, ensuring that stratification informs equity-driven interventions rather than labeling or penalizing vulnerable groups.

#### 1.7.5 Measurement caveats: feasibility, invariance, and the risk of over-quantification

Weighting and aggregation choices should themselves be treated as empirical questions rather than settled features of the framework. Unit-weighted composites are a defensible default until factor-analytically derived weights have been replicated across independent samples, and profile-based reporting (a five-dimension profile rather than a single composite score) may be preferable wherever composite scores would obscure meaningful within-person variation.

Several further caveats warrant emphasis. First, the full battery imposes non-trivial participant burden; staged screen-and-confirm designs and planned-missingness protocols can reduce testing time substantially. Second, missing data—particularly from wearables—are rarely missing at random and require principled handling (multiple imputation, full-information maximum likelihood, and sensitivity analyses). Third, cross-cultural comparability cannot be assumed: formal tests of measurement invariance (configural, metric, scalar) across language, age, sex, and socioeconomic groups are a precondition for any between-group comparison. Finally, over-quantification is itself a risk: converting recovery, sociality, and digital life into permanent metrics may induce surveillance fatigue and instrumentalize the very behaviors the framework seeks to support. Measurement should therefore be time-limited, purpose-bound, and paired with non-quantified, interpretive follow-up.

#### 1.7.6 Validation roadmap

A systematic validation program is essential to establish credibility and adoption of the paradigm:

- Reliability: Test–retest reliability for questionnaires, inter-device reliability for wearables, and assessment of practice effects for cognitive tasks.
- Validity: Evidence for convergent validity (e.g., physical fitness scores correlating with VO₂max), discriminant validity (low cross-loadings across dimensions), predictive validity (associations with disease incidence, sickness absence, or work performance), and fairness (measurement invariance across demographic groups).
- Feasibility: Practical considerations including participant burden, financial and logistical cost, accessibility, user experience, and compliance with data governance standards.

### 1.8 Implementation in practice: examples and use cases

#### 1.8.1 Clinical and community programs

A lifestyle clinic illustrates how the five dimensions of fitness can be integrated into patient intake and longitudinal follow-up. Beyond standard vitals and exercise testing, assessments include sleep quality, stress and resilience (CD-RISC), social network vulnerability (LSNS-6), work ability (WAI), and eHealth literacy (eHEALS). Based on these multidimensional profiles, patients receive personalized plans that combine progressive resistance training, autonomy-supportive coaching, referral to walking groups, structured recovery protocols (e.g., protected time, detachment skills), and device-agnostic digital health strategies with privacy-preserving settings and opt-in AI prompts. Outcomes are monitored quarterly, enabling adaptive goal-setting in response to life events. This model demonstrates the feasibility of embedding multidimensional fitness assessment into routine clinical and community care.

#### 1.8.2 Workplace transformation

A knowledge-work firm pilots an “occupational fitness” initiative to address employee health and performance. Using de-identified, aggregate data from validated surveys (JCQ, ERI, REQ), high-demand/low-control teams are identified and targeted for job redesign interventions. Changes include clarified roles, expanded decision latitude, meeting-free focus blocks, and a right-to-disconnect policy. Leadership training emphasizes sustainable performance and modeling recovery practices. An optional wearable program provides choice of devices (or none), transparent data use (with no individual surveillance), weekly activity credits, and social challenges that reward inclusion rather than competition. This case highlights how occupational fitness can be operationalized through structural and cultural reforms, rather than relying solely on individual-level behavior change.

#### 1.8.3 Municipal health equity

At the population level, a municipality implements a multidimensional fitness strategy by pairing built-environment modifications (safe crossings, shaded areas, improved lighting, mixed-use zoning) with socially anchored programming, such as walking school busses and intergenerational activity clubs, targeted to neighborhoods with the highest inactivity burden. Community health workers facilitate digital literacy workshops and provide access to wearables through lending programs at libraries, ensuring equity in digital engagement. Public dashboards report neighborhood-level progress without individual tracking, fostering transparency and accountability while respecting privacy. This example demonstrates how local governments can advance fitness as a public good by integrating physical, social, and digital resources within an equity framework.

### 1.9 Anticipating critiques

“This is just rebranding general well-being.”

The proposed paradigm is not a loose catch-all concept. Rather, it defines fitness as a multidimensional capacity—operationalizable, measurable, and trainable—comprising interacting subsystems that enable sustainable performance and health. Importantly, the construct is distinct from hedonic well-being and remains narrower than global quality of life. Whereas well-being frameworks emphasize subjective states, the fitness paradigm emphasizes functional capacities that can be systematically developed, assessed, and evaluated.

“It is too complex to measure.”

Complexity should not justify neglect. Many scientific domains (e.g., mental health, organizational psychology, social epidemiology) manage multi-level constructs with validated methods. The same applies here: multimethod batteries and staged assessments (e.g., screen–confirm strategies) can keep burden manageable. Moreover, numerous validated instruments already exist—including the Work Ability Index (WAI), Job Content Questionnaire (JCQ), Effort–Reward Imbalance (ERI) scale, Lubben Social Network Scale (LSNS-6), and eHealth Literacy Scale (eHEALS)—while sensor-based passive measures can further reduce respondent load when applied judiciously.

“Digital fitness privileges the affluent.”

This concern is valid. The paradigm explicitly addresses it through strategies such as public provisioning (e.g., lending libraries for devices), platform accessibility requirements, and non-digital pathways to equivalent outcomes. Moreover, digital fitness extends beyond device ownership: it encompasses the right to disconnect, to manage one’s data, and to refuse data collection. These principles position digital fitness as an equity-sensitive construct, not a privilege of the affluent.

“Evidence for AI coaching is nascent.”

Indeed, current evidence for AI-enabled coaching is preliminary and heterogeneous. This is precisely why the framework embeds ethical guardrails, transparency requirements, and human oversight mechanisms, along with a structured research agenda to test efficacy, safety, and equity across diverse populations. The goal is not to overstate the evidence but to acknowledge emerging opportunities while demanding rigorous evaluation before widespread adoption.

### 1.10 A 10-point research agenda

- Construct validation. Test hierarchical models of multidimensional fitness in diverse cohorts; evaluate measurement invariance across sex, age, culture, and socioeconomic strata.
- Causal mechanisms. Use longitudinal and experimental designs (including micro-randomized trials) to map directional links among dimensions (e.g., how recovery and social support amplify training response).
- Behavior change optimization. Compare COM-B and SDT-grounded strategies in pragmatic trials; identify moderators (identity, executive function, sleep status).
- Equity by design. Co-produce interventions with marginalized communities; evaluate accessibility, affordability, and cultural fit.
- Built-environment natural experiments. Leverage urban design changes to test population effects on movement, social interaction, and safety.
- Workplace policy trials. Randomize units to right-to-disconnect and workload-leveling policies; measure burnout, performance, and retention.
- Digital guardrails. Develop and validate AI-ethics checklists and algorithmic impact assessments specific to fitness contexts; assess privacy-by-design implementations.
- Lifespan trajectories. Model developmental windows where interventions yield outsized returns (e.g., transitions into school, workforce, parenthood, retirement).
- Economic evaluation. Cost-effectiveness of multidimensional vs. traditional programs; include productivity and social care offsets.
- Open science infrastructure. Shared ontologies, data standards, and repositories to harmonize measures and enable replication (Figure 6).

**Figure 6:**
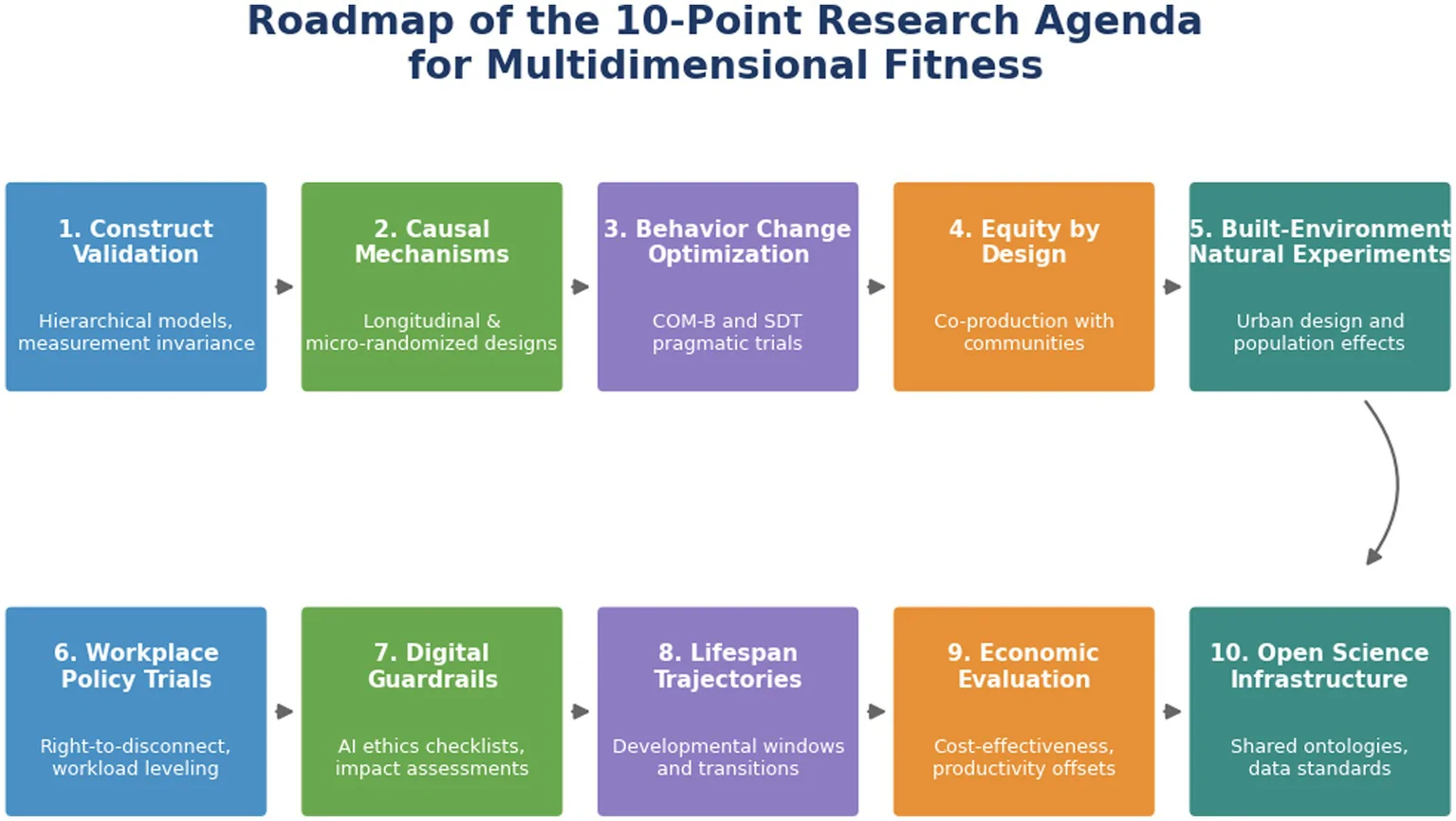
Ten-point research and implementation agenda for the multidimensional fitness paradigm. This framework outlines the sequential priorities required to operationalize and evaluate holistic fitness, including construct validation, causal mechanism testing, behavior change optimization, built environment interventions, workplace policy trials, digital ethics guardrails, economic evaluation, and open science infrastructure. Collectively, these components provide a roadmap for advancing evidence-based, equitable, and ethically governed fitness research and policy.

#### 1.10.1 Sequencing the agenda: a staged roadmap for validating and developing the model

These 10 priorities are neither equally urgent nor mutually independent: several presuppose the others, and pursuing them in parallel would produce intervention trials on instruments whose structure has not been established. Because the framework is a conceptual proposal rather than a validated instrument, its development should proceed in a defined sequence with explicit deliverables at each stage and pre-specified criteria for continuing, revising, or abandoning it. The indicative timelines below assume conventional funding cycles and are offered as a structure for coordination rather than as a schedule.

##### 1.10.1.1 Stage 1 — Specification and harmonization (0–12 months)

Deliverables: a published item bank mapping every candidate indicator to the specific mechanism it operationalizes; a formal Delphi consensus establishing the Tier 1 core set and its cut-points, following established core outcome set methodology and including the populations to be assessed among the panelists (50); translated and culturally adapted versions of the core instruments in at least four languages spanning distinct linguistic families; and a pre-registered measurement protocol with an open data dictionary and shared metadata standard. No new primary data collection is required at this stage; the immediate task is to harmonize instruments that already exist rather than to create new ones.

##### 1.10.1.2 Stage 2 — Structural and psychometric validation (12–30 months)

A cross-sectional study in a demographically diverse sample of at least 1,000 adults, powered for confirmatory factor analysis, testing the proposed higher-order structure against competing specifications: a single general factor, a three-factor biopsychosocial solution, and a bifactor model. This stage must also establish test–retest reliability, inter-device reliability for wearable-derived indicators, and configural, metric, and scalar invariance across sex, age, language, and socioeconomic strata (51). Discriminant validity against adjacent constructs—subjective wellbeing, health-related quality of life, and general self-rated health—must be demonstrated here; in its absence, the ‘rebranding’ objection addressed in Section 1.8 (Anticipating Critiques) stands unanswered.

##### 1.10.1.3 Stage 3 — Predictive and incremental validity (24–60 months)

Prospective follow-up, conducted wherever possible by appending the core set to ongoing cohorts rather than launching new ones, testing whether the five-dimension profile predicts hard outcomes: incident non-communicable disease, sickness absence, disability pension, decline in work ability, and all-cause mortality. The decisive analysis is incremental rather than overall association—whether the mental, social, occupational, and digital dimensions add predictive information beyond physical fitness and standard demographic and socioeconomic covariates. A dimension that does not is not earning its place in the model.

##### 1.10.1.4 Stage 4 — Intervention and optimization (36–72 months)

Once measurement is established, the framework’s causal claims become testable. Factorial and micro-randomized designs conducted within an optimization framework are better suited than two-arm trials to identifying which dimension-specific components contribute, for whom, and in what combination (52), while workplace and municipal policies are more appropriately evaluated through cluster-randomized designs and natural experiments. Economic evaluation should be embedded from the outset rather than appended at the end, since the case for a more expensive assessment model rests on demonstrating offsets in productivity, sickness absence, and social care.

##### 1.10.1.5 Stage 5 — Implementation, equity audit, and governance (ongoing)

Feasibility, acceptability, and completion rates in routine clinical, workplace, and municipal settings; algorithmic impact assessment and independent audit of any AI-enabled component; explicit evaluation of whether use of the framework narrows or widens measured inequities relative to standard care; and open repositories for instruments, analysis code, and de-identified data to permit replication and reanalysis.

##### 1.10.1.6 Falsifiability and stopping rules

A conceptual framework that cannot fail is of limited scientific value, and we therefore specify in advance what would count as disconfirmation. The framework should be substantially revised or abandoned if (a) the five-dimension structure does not fit better than more parsimonious alternatives across independent samples; (b) scalar invariance cannot be established across major demographic groups, since between-group comparison would then be uninterpretable and equity-sensitive reporting unsafe; (c) the non-physical dimensions add negligible incremental prediction of functional and health outcomes; or (d) dimension scores prove insensitive to interventions already known to change the underlying capacities. Digital fitness carries the highest risk of failing conditions (a) and (c), consistent with its status as the least mature dimension, and should therefore be the first subjected to these tests; if it fails them, the appropriate response is to demote it to a contextual determinant rather than to preserve it by relaxing the criteria set out in Section 1.3.8 (Conceptual Boundaries).

### 1.11 Policy implications and recommendations

Translating the Multidimensional Fitness Paradigm into actionable policy requires coordinated reforms across public health, education, workplace regulation, technology governance, and urban planning. Recent advances within this paradigm demonstrate that fragmented applications—such as digital tracking models, cognitive control and reserve training, job design and recovery strategies, and social integration programs—achieve the greatest impact when strategically integrated. Accordingly, a multimodal integration approach is proposed, unifying these previously independent components into a single, interactive ecosystem. This approach enables the creation of personalized, adaptive, ethically grounded, and sustainable fitness systems that operate across physical, cognitive, occupational, and social dimensions. Embedding such systemic integration into national and institutional policies can foster transdisciplinary coherence and long-term sustainability.

To avoid overstating the evidence, the recommendations below are explicitly graded. Recommendations concerning physical activity, sedentary behavior, and resistance training are evidence-based, consistent with WHO guidelines and meta-analytic evidence. Recommendations on job design, recovery, and right-to-disconnect policies combine established occupational-health evidence with expert opinion regarding implementation. Recommendations on AI governance, algorithmic auditing, and digital equity are primarily normative and precautionary, grounded in ethical frameworks rather than in outcome trials. Readers should weigh each recommendation accordingly.

#### 1.11.1 Public health

Align national guidelines with WHO (3) while broadening their scope to explicitly incorporate resistance training across all age groups, sedentary behavior reduction, social participation, recovery literacy, and digital literacy. Invest in community connectors, equitable access initiatives, and safe public spaces that promote inclusive physical and social engagement.

#### 1.11.2 Education and training

Embed multidimensional fitness concepts into curricula for coaches, clinicians, educators, and organizational leaders. Integrate behavioral science frameworks such as the COM-B model and Self-Determination Theory (SDT), alongside modules on recovery, emotion regulation, data rights, and digital hygiene to ensure cross-sectoral competence.

#### 1.11.3 Workplace regulation

Encourage or mandate “right-to-disconnect” policies, incentivize equitable job design that enhances decision latitude and fairness, and enforce transparent data governance for workplace wellness technologies. Ban any form of individual-level surveillance or coercive data use in employment contexts, ensuring that well-being initiatives remain voluntary and ethical.

#### 1.11.4 Technology governance

Require independent impact assessments, algorithmic bias testing, and clear informed consent procedures for all AI-enabled fitness applications. Promote interoperability and data portability standards, while supporting device-lending programs, subsidies for low-income users, and minimum accessibility requirements to reduce the digital divide.

#### 1.11.5 Urban planning

Recognize walkability, green infrastructure, public transit, and shade as essential health assets. Integrate community-based social programming and intergenerational activity design within urban renewal projects to maximize utilization and promote inclusive participation.

### 1.12 Limitations

This review has several limitations. First, it is a narrative and critical—not systematic—synthesis: despite broad multidisciplinary coverage, source selection was purposive, may be subject to selection bias, and involved no formal quality appraisal or meta-analytic pooling. Second, the multidimensional fitness framework remains largely conceptual. The proposed higher-order factor structure, indicator weights, and interpretive thresholds have not been empirically validated, and discriminant validity against adjacent constructs (wellbeing, quality of life, work ability) must be demonstrated rather than assumed. Third, the evidence base is uneven across dimensions—strong for physical and occupational constructs, emerging for mental and social fitness as integrated capacities, and largely propositional for digital fitness.

Fourth, most of the cited evidence originates from high-income, Western, industrialized contexts; cross-cultural generalizability and measurement invariance remain untested. Fifth, the proposed indicator set, although grounded in validated instruments, has not been examined as a battery with respect to feasibility, respondent burden, or redundancy. Finally, several policy recommendations extend beyond current outcome evidence and should be read as expert or precautionary positions pending empirical evaluation.

## 2 Conclusion

The framework advanced here should be read as a conceptual proposal—a hypothesis-generating map rather than a validated instrument—whose value will ultimately be determined by the empirical program outlined above. With that caveat, the adoption of a multidimensional fitness paradigm has the potential to reshape the trajectory of science, clinical practice, and policy over the next decade. For the scientific community, it invites a paradigm shift from reductionist models toward integrative frameworks that can more accurately capture the complexity of human functioning in real-world contexts. For clinical and rehabilitation practice, it promises more personalized, equitable, and ethically grounded interventions that move beyond physiology to include psychological resilience, social participation, occupational sustainability, and digital literacy as core determinants of health. At the policy level, embracing multidimensional fitness could catalyze reforms in public health guidelines, workplace regulations, and technology governance, aligning them with the realities of 21 first century life. Ultimately, positioning fitness as a biopsychosocio-digital capacity not only broadens the scope of prevention and intervention but also charts a forward-looking agenda in which individual flourishing, organizational resilience, and societal wellbeing are mutually reinforcing. Pending such empirical validation, this vision positions the multidimensional paradigm as a promising candidate framework—rather than an established standard—for guiding research, practice, and policy toward healthier, more adaptive, and more inclusive futures.

## References

[1] Ozemek C Bonikowske AR Christle JW Gallo PM. ACSM’S Guidelines for Exercise Testing and Prescription. 12th ed. Alphen aan den Rijn: Wolters Kluwer (2026).

[2] World Health Organization. Global Action Plan on Physical Activity 2018–2030: more Active People for a Healthier World. Geneva: World Health Organization (2019).

[3] World Health Organization. WHO Guidelines on Physical Activity and Sedentary Behaviour. Geneva: World Health Organization (2020). 33369898

[4] National Academy of Sports Medicine. NASM Essentials of Personal Fitness Training. 7th ed. Burlington, MA: Jones and Bartlett Learning (2021).

[5] Ekelund U Steene-Johannessen J Brown WJ Fagerland MW Owen N Powell KE . Does physical activity attenuate, or even eliminate, the detrimental association of sitting time with mortality? A harmonised meta-analysis of data from more than 1 million men and women. Lancet. (2016) 388:1302–10. doi: 10.1016/S0140-6736(16)30370-1, 27475271

[6] Garber CE Blissmer B Deschenes MR Franklin BA Lamonte MJ Lee IM . Quantity and quality of exercise for developing and maintaining cardiorespiratory, musculoskeletal, and neuromotor fitness in apparently healthy adults: guidance for prescribing exercise. Med Sci Sports Exerc. (2011) 43:1334–59. doi: 10.1249/MSS.0b013e318213fefb, 21694556

[7] Bauman AE Reis RS Sallis JF Wells JC Loos RJ Martin BW. Correlates of physical activity: why are some people physically active and others not? Lancet. (2012) 380:258–71. doi: 10.1016/S0140-6736(12)60735-1, 22818938

[8] Sallis JF Cerin E Conway TL Adams MA Frank LD Pratt M . Physical activity in relation to urban environments in 14 cities worldwide: a cross-sectional study. Lancet (London, England). (2016) 387:2207–17. doi: 10.1016/S0140-6736(15)01284-2, 27045735 PMC10833440

[9] Piwek L Ellis DA Andrews S Joinson A. The rise of consumer health wearables: promises and barriers. PLoS Med. (2016) 13:e1001953. doi: 10.1371/journal.pmed.1001953, 26836780 PMC4737495

[10] Topol E. Deep Medicine: How Artificial Intelligence can make Healthcare Human again. London: Hachette UK (2019).

[11] Deci EL Ryan RM. Self-determination theory: a macrotheory of human motivation, development, and health. Can Psychol. (2008) 49:182–5. doi: 10.1037/a0012801

[12] Diamond A. Executive functions. Annu Rev Psychol. (2013) 64:135–68. doi: 10.1146/annurev-psych-113011-143750, 23020641 PMC4084861

[13] Gross JJ. Emotion regulation: current status and future prospects. Psychol Inq. (2015) 26:1–26. doi: 10.1080/1047840x.2014.940781

[14] Teixeira PJ Carraça EV Markland D Silva MN Ryan RM. Exercise, physical activity, and self-determination theory: a systematic review. Int J Behav Nutr Phys Activity. (2012) 9:78. doi: 10.1186/1479-5868-9-78, 22726453 PMC3441783

[15] Holt-Lunstad J Smith TB Layton JB. Social relationships and mortality risk: a meta-analytic review. PLoS Med. (2010) 7:e1000316. doi: 10.1371/journal.pmed.1000316, 20668659 PMC2910600

[16] Umberson D Karas Montez J. Social relationships and health: a flashpoint for health policy. J Health Soc Behav. (2010) 51:S54–66. doi: 10.1177/0022146510383501, 20943583 PMC3150158

[17] Bowleg L. The problem with the phrase women and minorities: intersectionality—an important theoretical framework for public health. Am J Public Health. (2012) 102:1267–73. doi: 10.2105/AJPH.2012.300750, 22594719 PMC3477987

[18] Karasek R Theorell T. Healthy Work: Stress, Productivity, and the Reconstruction of Working Life. New York: Basic Books (1990).

[19] Maslach C Leiter MP. Understanding the burnout experience: recent research and its implications for psychiatry. World Psychiatry. (2016) 15:103–11. doi: 10.1002/wps.20311, 27265691 PMC4911781

[20] Siegrist J. Adverse health effects of high-effort/low-reward conditions. J Occup Health Psychol. (1996) 1:27–41. doi: 10.1037/1076-8998.1.1.27, 9547031

[21] Sonnentag S Fritz C. The recovery experience questionnaire: development and validation of a measure for assessing recuperation and unwinding from work. J Occup Health Psychol. (2007) 12:204–21. doi: 10.1037/1076-8998.12.3.204, 17638488

[22] Floridi L Cowls J Beltrametti M Chatila R Chazerand P Dignum V . AI4People-an ethical framework for a good AI society: opportunities, risks, principles, and recommendations. Mind Mach. (2018) 28:689–707. doi: 10.1007/s11023-018-9482-5, 30930541 PMC6404626

[23] Vandemeulebroucke T. The ethics of artificial intelligence systems in healthcare and medicine: from a local to a global perspective, and back. Pflugers Archiv. (2025) 477:591–601. doi: 10.1007/s00424-024-02984-3, 38969841 PMC11958494

[24] Guthold R Stevens GA Riley LM Bull FC. Worldwide trends in insufficient physical activity from 2001 to 2016: a pooled analysis of 358 population-based surveys with 1·9 million participants. Lancet Glob Health. (2018) 6:e1077–86. doi: 10.1016/S2214-109X(18)30357-7, 30193830

[25] Guthold R Stevens GA Riley LM Bull FC. Global trends in insufficient physical activity among adolescents: a pooled analysis of 298 population-based surveys with 1·6 million participants. The Lancet Child & adolescent health. (2020) 4:23–35. doi: 10.1016/S2352-4642(19)30323-2, 31761562 PMC6919336

[26] Ding D Lawson KD Kolbe-Alexander TL Finkelstein EA Katzmarzyk PT van Mechelen W . The economic burden of physical inactivity: a global analysis of major non-communicable diseases. Lancet. (2016) 388:1311–24. doi: 10.1016/S0140-6736(16)30383-X, 27475266

[27] Eysenbach G. The law of attrition. J Med Internet Res. (2005) 7:e11. doi: 10.2196/jmir.7.1.e11, 15829473 PMC1550631

[28] Depper A Howe PD. Are we fit yet? English adolescent girls’ experiences of health and fitness apps. Health Sociol Rev. (2017) 26:98–112. doi: 10.1080/14461242.2016.1196599

[29] Van Dijk JA. Digital divide research, achievements and shortcomings. Poetics. (2006) 34:221–35. doi: 10.1016/j.poetic.2006.05.004

[30] Bickerdike L Booth A Wilson PM Farley K Wright K. Social prescribing: less rhetoric and more reality. A systematic review of the evidence. BMJ Open. (2017) 7:e013384. doi: 10.1136/bmjopen-2016-013384, 28389486 PMC5558801

[31] King AC Stokols D Talen E Brassington GS Killingsworth R. Theoretical approaches to the promotion of physical activity: forging a transdisciplinary paradigm. Am J Prev Med. (2002) 23:15–25. doi: 10.1016/s0749-3797(02)00470-1, 12133734

[32] Michie S Van Stralen MM West R. The behaviour change wheel: a new method for characterising and designing behaviour change interventions. Implement Sci. (2011) 6:42. doi: 10.1186/1748-5908-6-42, 21513547 PMC3096582

[33] Friedman NP Miyake A. Unity and diversity of executive functions: individual differences as a window on cognitive structure. Cortex. (2017) 86:186–204. doi: 10.1016/j.cortex.2016.04.023, 27251123 PMC5104682

[34] Miyake A Friedman NP Emerson MJ Witzki AH Howerter A Wager TD. The unity and diversity of executive functions and their contributions to complex "frontal lobe" tasks: a latent variable analysis. Cogn Psychol. (2000) 41:49–100. doi: 10.1006/cogp.1999.0734, 10945922

[35] Connor KM Davidson JR. Development of a new resilience scale: the Connor-Davidson resilience scale (CD-RISC). Depress Anxiety. (2003) 18:76–82. doi: 10.1002/da.10113, 12964174

[36] Jetten J Haslam C Alexander SH. The Social Cure: Identity, Health and Well-Being. Hove: Psychology Press (2012).

[37] Sonnentag S Cheng BH Parker SL. Recovery from work: advancing the field toward the future. Annu Rev Organ Psychol Organ Behav. (2022) 9:33–60. doi: 10.1146/annurev-orgpsych-012420-091355

[38] Norman CD Skinner HA. eHEALS: the eHealth literacy scale. J Med Internet Res. (2006) 8:e27. doi: 10.2196/jmir.8.4.e27, 17213046 PMC1794004

[39] Gill DL Williams L Reifsteck EJ. Psychological Dynamics of sport and Exercise. Champaign, IL: Human Kinetics (2017).

[40] Roberts GC Treasure D. Advances in Motivation in sport and Exercise. Champaign, IL: Human Kinetics (2012).

[41] Salmon P. Effects of physical exercise on anxiety, depression, and sensitivity to stress: a unifying theory. Clin Psychol Rev. (2001) 21:33–61. doi: 10.1016/S0272-7358(99)00032-X, 11148895

[42] Venkatesh V Morris MG Davis GB Davis FD. User acceptance of information technology: toward a unified view. MIS Q. (2003) 27:425–78. doi: 10.2307/30036540

[43] Hampton KN. Studying the digital: directions and challenges for digital methods. Annu Rev Sociol. (2017) 43:167–88. doi: 10.1146/annurev-soc-060116-053505

[44] Gupta N Jilla S. Digital Fitness Connector: Smart Wearable System. In: *2011 First International Conference on Informatics and Computational Intelligence*, pp. 118–121. (2011).

[45] Gulati AK Lobo RE N N Bhat V Bora N K V . Young adults journey with digital fitness tools-a qualitative study on use of fitness tracking device. F1000Res. (2024) 13:1296. doi: 10.12688/f1000research.158037.1, 39534658 PMC11555328

[46] Kossowski J Lenz A Heumüller E Richter S. *Digital Fitness–The goal of digital Transformation*. (2020).

[47] Leong DP Teo KK Rangarajan S Lopez-Jaramillo P Avezum A Orlandini A . Prognostic value of grip strength: findings from the prospective urban rural epidemiology (PURE) study. Lancet. (2015) 386:266–73. doi: 10.1016/S0140-6736(14)62000-6, 25982160

[48] Hughes ME Waite LJ Hawkley LC Cacioppo JT. A short scale for measuring loneliness in large surveys: results from two population-based studies. Res Aging. (2004) 26:655–72. doi: 10.1177/0164027504268574, 18504506 PMC2394670

[49] Ilmarinen J. Work ability-a comprehensive concept for occupational health research and prevention. Scand J Work Environ Health. (2009) 35:1–5. doi: 10.5271/sjweh.1304, 19277432

[50] Williamson PR Altman DG Bagley H Barnes KL Blazeby JM Brookes ST . The COMET handbook: version 1.0. Trials. (2017) 18:280. doi: 10.1186/s13063-017-1978-4 PMC5499094 28681707

[51] Putnick DL Bornstein MH. Measurement invariance conventions and reporting: the state of the art and future directions for psychological research. Dev Rev. (2016) 41:71–90. doi: 10.1016/j.dr.2016.06.004, 27942093 PMC5145197

[52] Collins LM. Optimization of Behavioral, Biobehavioral, and Biomedical Interventions: The Multiphase Optimization Strategy (MOST). Berlin: Springer (2018).

